# Molecular Constraints of Sperm Sex Sorting via TLR7/8 Activation

**DOI:** 10.3390/ani15202976

**Published:** 2025-10-14

**Authors:** Alikhan Magauiya, Kausar Torebek, Filipp Georgijevič Savvulidi, Martin Ptáček, Christopher LeBrun, Lucie Langerová, Elbosyn Sagdat, Saltanat Baikoshkarova, Nurlan Malmakov

**Affiliations:** 1 Meat Sheep Breeding Department, Kazakh Research Institute of Livestock and Fodder Production, Almaty 050 035, Kazakhstan; orda.ezhenid@gmail.com (A.M.); torebekkausar@gmail.com (K.T.); elbosyn.sagdat.92@mail.ru (E.S.); nurlan_malmakov@mail.ru (N.M.); 2 Ecomed-Shymkent, Shymkent 160 023, Kazakhstan; 3 Department of Animal Science, Faculty of Agrobiology, Food and Natural Resources, Czech University of Life Sciences (CULS), 165 00 Prague, Czech Republic; ptacekm@af.czu.cz (M.P.); xlebc001@studenti.czu.cz (C.L.); langerova@af.czu.cz (L.L.); 4 Ecomed, Almaty 050 009, Kazakhstan; ecomed2017@mail.ru; 5 New Generation Clinic, Almaty A15E2H1, Kazakhstan

**Keywords:** livestock, sperm sexing, activation of TLR7/8, R848, sperm motility, species-specific differences, murine, caprine, ovine, bovine, swine, canine, artificial insemination

## Abstract

**Simple Summary:**

Sperm sex selection is important in livestock for economic reasons. The current method, Fluorescence-Activated Cell Sorting (FACS), is costly and can harm sperm. A newer, cheaper method uses TLR7/8 receptors to slow down X-bearing sperm, allowing Y-bearing sperm to be separated using the swim-up technique. This method works well in murine, caprine, ovine, and bovine species, but not in swine and canine species due to species-specific differences. This review explores the possible reasons behind those differences.

**Abstract:**

In modern livestock, the demand for sperm sex selection technologies is high, as the ability to deliberately produce offspring of a specific sex offers significant economic advantages. Traditionally, sperm sorting is performed using Fluorescence-Activated Cell Sorting. However, the flow cytometric method is expensive, technically complex, and associated with reduced sperm viability. An alternative promising method involves the use of Toll-like TLR7/8 receptors for the selective binding of spermatozoa of a particular sex. It was discovered previously that the activation of TLR7/8 by its ligand(s) selectively inhibits the motility of X-bearing sperm without affecting the motility of Y-bearing sperm. The swim-up technique, which separates sperm based on sex chromosome type by isolating fractions enriched in either X- or Y-bearing gametes due to differences in their motility, can be used with this method. Sperm sex sorting via the TLR7/8 activation is cheap, technically non-complex, and does not affect sperm viability negatively. The goal of this review is to provide an overview of the TLR7/8-dependent sperm sorting method. Further, we discuss why the method of sperm sorting via TLR7/8 activation is successfully implemented in some animal species (such as murine, caprine, ovine, and bovine) but fails in others, like swine and canine.

## 1. Introduction

Sperm sex sorting is a relevant challenge in assisted reproductive technologies (ART) for both human and animal reproduction. The primary medical application of this technology lies in the prevention of X-linked hereditary diseases such as Duchenne muscular dystrophy, hemophilia, and other genetic disorders that predominantly affect male offspring, as reviewed in [1]. The use of sex selection methods in medical practices provides couples with a high risk of having a child with the aforementioned conditions the opportunity to conceive a child of the desired sex. This reduces the need for pregnancy termination following the detection of genetic disorders during prenatal screening. As a result, this technology contributes to the birth of healthy offspring and lessens the psychological and ethical burden associated with making such difficult reproductive decisions.

In livestock breeding, the demand for sex selection technologies is particularly high, as the ability to deliberately produce offspring of a specific sex offers significant economic advantages. For example, male animals are generally more desirable in meat production, while females are preferred in dairy farming.

Traditionally, sperm sorting is performed using FACS, which is based on differences in DNA content between X- and Y-bearing sperm. However, this method is expensive, technically complex, and associated with reduced sperm viability. Another serious limitation of the semen FACS sorting protocol is sire-to-sire variability. While most bulls can be successfully semen-sexed, some consistently show lower pregnancy rates after sorting. Certain miRNAs (miR-34c, miR-7859, and miR-342) found in conventionally produced semen have been linked to the fertilizing potential of bovine sperm after sex-sorting [2]. As a result, active research is underway to develop alternative approaches [3].

The evolution of sperm sexing techniques encompasses a wide range of technologies from density gradient separation methods to immunological approaches. Such innovative approaches have the potential to improve the efficiency and accessibility of the procedure, making them promising for both veterinary applications and ART. Additionally, the advancement of sperm sorting technologies opens new opportunities for both medicine and agriculture, reducing the risk of hereditary diseases in humans and enhancing the productivity of livestock farming operations. One promising direction is a method developed by Umehara et al. [4], which involves the use of specific molecular markers for the selective binding of spermatozoa of a particular sex. They discovered that the expression of Toll-like receptors TLR7 and TLR8, which are encoded by the X chromosome (Table 1), underlies motility differences between X- and Y-bearing sperm. Their study demonstrated that these receptors are expressed in approximately 50% round spermatids and epididymal spermatozoa, with TLR7 localized in the tail and TLR8 in the midpiece of the sperm cell. Activation of TLR7/8 selectively reduced the motility of X-bearing sperm without affecting Y-bearing sperm. More importantly, this process did not compromise cell viability or acrosome formation [4].

## 2. Sperm Sex Sorting via the TLR7/8 Receptor Activation Method

### 2.1. Activation of TLR7/8

Resiquimod (R848) is a low-molecular-weight imidazoquinoline compound that acts as an agonist of TLR7/8 and is currently widely used in clinical practice for the treatment of viral infections and cancers due to its ability to modulate the innate immune response [7]. In the studies conducted by Umehara et al. [4], it was found that activation of TLR7/8 through R848 exerts a differential effect on X- and Y-bearing sperm, influencing their energy metabolism and motility. In Y-bearing sperm, both mitochondrial and glycolytic pathways of ATP generation function independently and are not affected by TLR7/8 activation. As a result, Y-sperm maintain high velocity and progressive motility even in the presence of R848.

**Figure 1:**
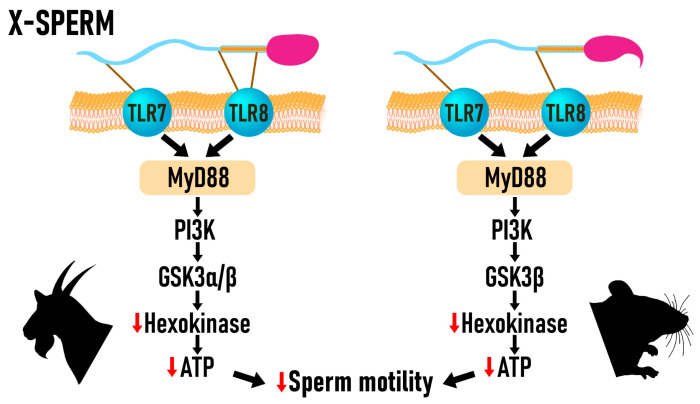
A diagram illustrating the activation of the TLR7/8 pathway in spermatozoa. In X-sperm, activation of TLR7/8 induces distinct signaling cascades that suppress ATP production and thereby reduce motility. In goats, TLR7/8, localized in the sperm tail, phosphorylates NFκB and GSK3α/β through a PI3K/AKT-independent pathway, leading to inhibition of hexokinase activity and decreased glycolytic ATP generation. In mice, TLR8 localized in the midpiece suppresses mitochondrial ATP production, while TLR7 in the sperm tail phosphorylates NFκB and GSK3α/β, further suppressing glycolysis. As a result, in both species, X-sperm exposed to TLR7/8 ligands exhibit reduced ATP availability and diminished sperm motility.

Notably, in X-bearing sperm, TLR7/8 activation has a stronger impact on energy metabolism. Activation of TLR8 leads to the suppression of mitochondrial ATP synthesis, thereby limiting the energy supply necessary for motility. Simultaneously, activation of TLR7 induces phosphorylation of key signaling molecules NFκB and GSK3α/β, which are associated with the inhibition of hexokinase activity, the enzyme responsible for catalyzing the first step of glycolysis. Collectively, these processes result in a significant reduction in ATP levels in X-bearing sperm, correlating with a marked decrease in their motility (Figure 1). Thus, activation of TLR7/8 via R848 selectively inhibits the motility of X-bearing sperm without affecting the kinetic parameters of Y-bearing sperm.

### 2.2. Swim-Up Technique to Separate Sperm Based on Sex Chromosome Type

In their study, Umehara et al. [4] used the swim-up method to separate sperm based on sex chromosome type, isolating fractions enriched in either X- or Y-bearing gametes based on differences in their motility. Subsequent analysis of the functional activity of treated sperm demonstrated that R848 did not have a negative impact on their fertilization capacity. After in vitro fertilization, the rate of pronucleus formation and the percentage of embryos reaching the blastocyst stage were comparable between the control group and the groups treated with R848. However, the composition of the resulting embryos differed significantly depending on the sperm fraction used. When using the upper fraction, predominantly containing Y-chromosome-bearing sperm, 89.6% of the resulting embryos had an XY genotype, while only 10.3% were XX. Conversely, fertilization with the lower fraction, primarily X-chromosome-bearing sperm, resulted in 69.8% XX embryos and 30.1% XY embryos. The ensuing embryo transfer into recipient females confirmed the ability to regulate offspring sex ratios: 83.1% of the born pups in the upper fraction group were male, whereas in the lower fraction group, 81.4% of the offspring were female [4]. Furthermore, a study by Ren et al. [5] demonstrated that the use of R848 sex-sorted sperm enabled efficient in vitro fertilization, with the proportion of female embryos reaching 80.52 ± 6.75%. This confirms the potential application of this method for selective fertilization of oocytes by X- or Y-bearing spermatozoa.

Analysis of the effects of R848 on the functional status of spermatozoa showed that as incubation time in a medium containing 1 µmol/L R848 increased, there was a decrease in mitochondrial activity and intracellular ATP levels, which correlated with reduced sperm motility. These findings indicate that R848 is capable of selectively modulating sperm motility through activation of the TLR7/8 signaling pathway in vitro [5].

### 2.3. Sperm Motility and ATP Generation

Spermatozoa possess two primary mechanisms for ATP generation that support their motility and fertilization capacity: glycolysis and the mitochondrial electron transport chain. The mitochondrial pathway operates in the midpiece of the sperm, where mitochondria are located, and plays a key role in maintaining linear progressive motility [8], essential for sperm transit through the uterus and fallopian tubes [9]. Meanwhile, the glycolytic pathway is localized in the tail region of the sperm and is activated during capacitation, which is a complex set of biochemical changes that prepare the sperm for fusion with the oocyte. Glycolytic ATP production predominantly supports hyperactivated motility, characterized by increased amplitude of tail beats and a zigzag movement pattern, both of which are necessary for penetrating the cumulus–oocyte complex and zona pellucida.

As sperm progress through the female reproductive tract, they initially rely on mitochondrial ATP production to sustain linear motility. As they near the oocyte, there is an irreversible shift from mitochondrial to glycolytic ATP generation, resulting in enhanced lateral tail movements and a transition to hyperactivated motility [10]. This metabolic shift is important to consider when developing sperm-sorting methods. In the presence of R848, Y-bearing sperm exhibit hyperactivated motility characterized by a zigzag movement pattern and tend to concentrate in the upper fraction during swim-up sorting, whereas X-bearing sperm sediment in the lower fraction due to a significant reduction in motility. After removal of R848 and washing, sperm motility is restored, allowing the use of sorted sperm for in vitro fertilization (IVF). However, applying this approach to artificial insemination (AI) may present challenges related to the recovery of motility and its impact on sperm viability in vivo.

### 2.4. Sperm Motility and Glucose Concentration

Later, Umehara et al. [11] demonstrated that increasing the glucose concentration in the culture medium alters sperm motility from linear to zigzag patterns. This type of motility, known as hyperactivation, facilitates the migration of highly motile sperm to the upper layer of the medium against the force of gravity. In their study, optimal glucose concentration was established for efficient separation of X- and Y-bearing sperm using R848. Murine sperms were incubated in media containing 0, 2, or 10 mM glucose for 60 min, after which the number of cells in the upper layer was quantified. The proportion of sperm concentrated in the upper layer increased to approximately 50% of the control value for the medium with 2 mM glucose, whereas with 0 or 10 mM glucose, this proportion did not exceed 10%. Furthermore, the addition of R848 to the medium containing 2 mM glucose significantly reduced the number of sperm in the upper layer, decreasing this value by about half.

The analysis of motility parameters showed that, in glucose-free medium, R848 did not have a statistically significant effect on sperm linear velocity or average movement speed. However, in the presence of 2 mM glucose and R848, both parameters were significantly reduced, indicating the impact of R848 on sperm motility regulatory mechanisms when the glycolytic pathway is active. Consistent with previous findings by Umehara et al. [4], the incubation of murine sperm in mHTF medium containing 2 mM glucose, followed by treatment with 0.3 µM R848 for 60 min, resulted in over 90% of sperm collected from the upper layer carrying the Y chromosome.

As an additional step, 500 µM creatine can be added to the sperm separation medium to enhance the method’s efficiency. It is known that creatine boosts ATP production via the glycolytic pathway and promotes hyperactivated sperm motility [12]. The addition of creatine led to a significant increase in the proportion of sperm concentrated in the upper layer compared to the control. Introducing R848 into the creatine-containing medium reduced this proportion by approximately half. Motility analysis revealed that among the groups treated with R848, the percentage of sperm rising to the upper layer in the presence of creatine was twice as high as in the medium without creatine. Further evaluation of fertilization efficiency demonstrated that sperm sorted in medium containing both R848 and creatine resulted in a significantly higher fertilization rate compared to sperm treated with R848 alone (83.2 ± 1.6% vs. 64.4 ± 3.2%). The proportion of XY embryos among zygotes derived from sperm treated with both creatine and R848 was 92.4 ± 4.2%. Thus, the use of creatine in a medium with 2 mM glucose and R848, followed by removal of R848, enabled the development of a method for producing embryos, predominantly with an XY karyotype, suitable for application in IVF programs. However, this approach has limitations for artificial insemination (AI). Sperm generate ATP through two main pathways: mitochondrial and glycolytic. The former sustains linear motility required for uterine transit, while the latter induces hyperactivated, zigzag motility near the oocyte. This metabolic shift is irreversible in vitro. In R848-based sorting under 2 mM glucose, Y-bearing sperm accumulated in the upper fraction, and X-bearing sperm sedimented. After washing, motility was restored, making the method suitable for IVF. In AI, however, recovery of motility and maintenance of sperm viability in vivo remain major challenges, restricting its direct application [11].

Nevertheless, there is evidence supporting the use of this approach in AI. In dairy goats inseminated with lower-fraction sperm, embryos were collected from the uteri of the does that conceived. Collected embryos were predominantly with an XX karyotype (88.9%). Although the number of embryos was limited, these findings highlight the potential of this technology for AI [5]. It should be noted that only a few studies have implemented the direct sperm–ligand strategy without alkaline treatment or other modifying factors that may influence the separation outcome.

### 2.5. Other Factors Affecting the Efficiency of the Method

Certain components, such as heparin or caffeine, can modulate the sperm response to R848 due to differences in metabolic conditions. In particular, seminal plasma contains capacitation-inhibiting factors, including cholesterol, which help maintain linear motility in ejaculated sperm. Consequently, sperm obtained directly from the ejaculate exhibit high mitochondrial metabolic activity alongside low glycolytic activity. Moreover, in cryopreserved sperm, capacitation status can be induced during the thawing process as a result of increased intracellular calcium concentration [13]. Collectively, these findings confirm that sperm with high glycolytic pathway activity predominantly migrate to the upper fraction during sperm sex-sorting methods in both murine and bovine.

Adapting this method for use with ejaculated sperm requires the development of a medium that increases the proportion of sperm rising to the upper fraction. It is important to consider that cryopreserved sperm exhibit altered membrane permeability due to cryodamage, distinguishing them from fresh sperm. Epididymal sperm membranes are known to have high permeability because they are protected by proteins and fatty acids present in seminal plasma. These differences may affect the R848 penetration into the cell and its biological effects. Therefore, when applying this method to ejaculated sperm, it is necessary to optimize R848 concentration, exposure duration, and culture medium composition, while taking into account the metabolic characteristics of spermatozoa from different origins [11].

In the study by Hou et al. [14], the efficacy of two alternative TLR7/8 ligands, double-stranded RNA-40 (dsRNA-40) and double-stranded RNA-DR (dsRNA-DR), was evaluated for the separation of X- and Y-chromosome-bearing murine spermatozoa and the subsequent production of embryos with altered sex ratios during IVF. The results demonstrated that modifying dsRNA molecules by attaching cholesterol to their 3′-end significantly enhanced their transfection efficiency into cells, which is consistent with a study conducted by Salim et al. [15]. This modification did not interfere with the binding of dsRNA-40 and dsRNA-DR to TLR7/8 receptors. Incubation of X-chromosome-bearing spermatozoa expressing TLR7/8 with dsRNA-40 and dsRNA-DR led to specific inhibition of their motility. This was accompanied by a decrease in ATP levels and a suppression of mitochondrial activity in the cells, indicating that the action of these ligands is mediated through the downstream GSK3α/β–hexokinase signaling pathway.

The use of spermatozoa from the upper layer of the medium after preliminary separation with dsRNA-40 or dsRNA-DR, which predominantly contained highly motile Y-bearing sperm, during IVF resulted in a significant shift in the sex ratio of embryos toward males. In the dsRNA-treated groups, the proportion of embryos with a male karyotype ranged from 65.90% to 74.93%.

The efficiency of X- and Y-sperm separation using dsRNA-40 and dsRNA-DR was found to be lower compared to that achieved with R848. This is likely due to differences in binding affinity between dsRNA and R848 to TLR7/8, as dsRNAs are weaker agonists of these receptors [14]. Another possible reason for the variation in effectiveness is related to the stability of the compounds: dsRNAs degrade rapidly [16], whereas R848 is a more stable molecule, prolonging its activity in the medium [17].

To optimize sperm sorting methods, various approaches have been investigated, including the use of alkaline diluents and TLR agonists. Studies by He et al. [18,19] found that incubating sperm at pH 7.4 for 40 min increased the proportion of Y-bearing sperm in the upper layer, which was significantly higher than the control group. Huang et al. [20], building on this method, investigated the combined effects of an alkaline diluent and R848. They demonstrated that a medium with pH 7.4 enhances the motility of Y-bearing sperm while reducing the motility of X-bearing sperm, whereas R848 selectively suppresses X-sperm without affecting Y-sperm. The combined application of these factors amplified the motility differences between the two sperm populations. Their study also identified the minimum effective concentration of R848 (pH 7.4) that allows for efficient sperm sorting without compromising fertilization capacity. Conversely, several studies reported conflicting results [21], indicating the possible influence of additional factors, such as experimental conditions, species-specific biological characteristics, and variability in sperm response to changes in medium pH.

## 3. Application of Sperm Sorting Based on the TLR7/8 Receptor Activation Method in Different Mammalian Species

### 3.1. Species with Successful Application of the TLR7/8 Receptor Activation Method

To date, the efficacy of TLR7/8-dependent sperm sorting has been well demonstrated in murine, bovine, caprine, and ovine (Table 2).

### 3.2. Species with Unsuccessful Application of the TLR7/8 Receptor Activation Method

On the other hand, the inefficacy of TLR7/8-dependent sperm sorting has been demonstrated in swine and canine (Table 3).

#### 3.2.1. Swine

Wu et al. [23] were the first to report the application of sperm sorting based on TLR7/8 activation in boars. In their study, a detailed localization of TLR7 and TLR8 expressions in germ cells was conducted. In boar testicular tissue, TLR7 and TLR8 were predominantly expressed in spermatocytes, round spermatids, and mature spermatozoa. Immunocytochemical analysis revealed that the TLR7 protein is primarily localized in the sperm head, with lesser expression in the tail, whereas TLR8 is mainly expressed in the tail region. The authors noted that the expression pattern of TLR7/8 in boars differs significantly from that observed in mice and cattle. Specifically, unlike previously studied species, pigs showed no significant differences in TLR7/8 expression between X- and Y-bearing spermatozoa. As a result, although the application of the R848 reduced overall sperm motility, it did not result in selective inhibition of X-bearing sperm. This indicates that effective sex-based sperm sorting in boars via TLR7/8 activation is not feasible and calls into question the applicability of this approach in pigs.

#### 3.2.2. Canine

Pan et al. [6] investigated the effect of R848 on canine sperm sorting and demonstrated that the levels of NFκB and GSK3α/β phosphorylation were significantly elevated in the R848-treated group compared to the control. Consistent with the findings of Umehara et al. [4], R848 activated the TLR7/8 signaling pathway, thereby promoting the phosphorylation of NFκB and GSK3α/β. Moreover, R848 reduced ATP levels in X-bearing spermatozoa via the GSK3α/β–hexokinase pathway, resulting in decreased motility of these cells.

However, unlike in other species, canine spermatozoa exhibit specific patterns of TLR7/8 expression in X- and Y-bearing cells: TLR8 is not expressed in spermatozoa, while TLR7 is detected in all sperm cells, localized in both the head and tail regions, regardless of whether it is an X- or Y-bearing sperm. Consequently, even though R848 is capable of inhibiting canine sperm motility via phosphorylation of NFκB and GSK3α/β, its application does not lead to the separation of X- and Y-chromosome-bearing spermatozoa. Thus, the use of R848 for canine sperm sorting proved to be ineffective, indicating a species-specific mechanism of TLR7/8 activation. These findings underscore the need to develop alternative approaches for sex control in dogs.

## 4. Discussion

According to Umehara et al., 2020 [11], the sperm-sorting method based on TLR7/8 activation (using R848) relies on functional differences between X- and Y-bearing sperm, which manifest during activation of the glycolytic pathway, a component of the capacitation process. The main limitations of this method, especially regarding capacitated Y-sperm in the upper fraction, relate less to cryopreservation after capacitation and more to the inability to use this sperm for AI. The known constraints related to the state of sperm (Y-sperm in the upper fraction) after separation include irreversibility of capacitation and changes in motility: sperm separation with R848 occurs when the glycolytic pathway is activated (using 2 mM glucose). The highly motile Y-sperms that migrate into the upper fraction exhibit zigzag motility (hyperactivation). This metabolic shift from the mitochondrial to the glycolytic pathway is irreversible, at least under in vitro conditions. The capacitation-based mechanism creates serious challenges for adapting this method to artificial insemination. For successful fertilization in the oviduct, sperm must initially rely on linear motility (supported by the mitochondrial pathway) within the uterus. Although the sources describe in detail how separated Y-sperm (from the upper fraction) can be successfully used directly for IVF (after removal of R848), since they recover hypermotility, no data are provided regarding their viability or the limitations of cryopreservation once sperm have been separated (and therefore capacitated/hyperactivated). It should also be noted that in the cattle protocol, already cryopreserved and thawed bull sperm are used to initiate the separation process. In thawed sperm, capacitation status is induced during the thawing process by calcium uptake, which contributes to the high level of glycolytic pathway activation required for the R848-based separation method.

The specificity of TLR expression in mice germ cells indicates their involvement not only in immune responses but also in the regulation of key stages of spermatogenesis. According to morpho-functional studies by Doğan et al. [24], TLR7 is exclusively expressed during the spermiogenesis stage, localized in elongated spermatids. These findings are consistent with observations by Umehara et al. [4], who demonstrated the role of TLR7 in the functional regulation of X-sperm motility, emphasizing its involvement in processes such as mitochondrial activity regulation, ATP production, and motility control occurring at the final stage of male germ cell differentiation.

Furthermore, the expression patterns of other TLRs also exhibit pronounced cell type- and stage-specific selectivity. TLR1 is detected in spermatocytes as well as in round and elongated spermatids; TLR2, TLR4, TLR7, and TLR13 are found exclusively in elongated spermatids. TLR3, TLR5, TLR11, and TLR12 are expressed in spermatocytes, round spermatids, and elongated spermatids, whereas spermatogonia express only TLR11. Such fine spatial and temporal regulation suggests that these receptors participate in distinct phases of proliferation, growth, and maturation of germ cells.

The functional role of TLRs in germ cells continues to be actively investigated. Beyond their involvement in innate immune responses, accumulating evidence indicates that these receptors participate in processes of morphogenesis, cellular remodeling, and maturation of spermatogenic cells. TLRs are synthesized in the endoplasmic reticulum, transported through the Golgi apparatus, and are subsequently either expressed on the cell surface (TLR1–6, TLR10) or retained within endosomal and/or lysosomal compartments (TLR3, TLR7, TLR8, TLR9, TLR11, TLR12, TLR13). In germ cells, particularly elongated spermatids, TLRs localize to endosomal compartments, which may reflect their involvement in the formation and functional modification of the acrosome, an organelle partly derived from endosomal structures.

It is known that TLR activation can trigger phagosome maturation and autophagic processes. These mechanisms are critical for the removal of defective or excess components in differentiating cells, including mitochondria. Selective degradation of mitochondria, also called mitophagy, represents a key step in the final stage of spermatogenesis, contributing to the energetic remodeling and morphological maturation of spermatozoa [6].

Zhu et al. [25] reported similar findings of decreased functional activity of spermatozoa upon activation of various TLRs using their respective agonists, such as LPS (TLR4), Pam3CSK4 (TLR1/2), Zymosan and PGN-SA (TLR2), FLA-ST (TLR5), R848 (TLR7/8), and CpG-DNA (TLR9). Incubation of both murine and human spermatozoa with these compounds resulted in a significant reduction in progressive motility (up to 74%), while cell viability remained unaffected. These data suggest that the effect is mediated not by cytotoxicity, but through functional regulation.

It has been demonstrated that activation of TLRs (except for TLR3) initiates an intracellular signaling cascade involving the adaptor protein MyD88, phosphatidylinositol 3-kinase (PI3K), and glycogen synthase kinase 3 alpha (GSK3α). This pathway affects mitochondrial activity in spermatozoa by reducing ATP levels and mitochondrial membrane potential, leading to energy depletion and a subsequent decline in motility. Under conditions of decreased mitochondrial membrane potential, ATP synthase activity shifts towards ATP hydrolysis, thereby exacerbating the energy deficit [25].

A distinctive feature of mature spermatozoa is the absence of NF-κB transcriptional pathway activation in response to TLR stimulation. This aligns with their biological nature, which is characterized by a high degree of chromatin condensation and minimal transcriptional and translational activity, precluding the classical immune response at the gene expression level. Thus, activation of TLR signaling in spermatozoa of mice and humans, regardless of the receptor type, leads to disruption of mitochondrial bioenergetics and reduced sperm motility.

A key question facing researchers is why the method of sperm sorting via TLR7/8 activation is successfully implemented in some animal species (such as murine, caprine, ovine, and bovine) but fails in others, like swine and canine. One possible explanation lies in the mechanisms governing the distribution of genetic material among haploid spermatid cells.

It is known that during spermatogenesis, spermatids are connected by cytoplasmic bridges through which mRNAs, proteins, and other molecules are exchanged, ensuring phenotypic equivalence among developing haploid cells. However, as demonstrated by Bhutani et al. [26], there are numerous genes whose products are unevenly distributed across these bridges. These genes have been designated as “genoinformational markers” (GIMs). They exhibit the ability to evade complete exchange between cells and can behave as selfish genetic elements, influencing the allelic composition in the offspring of species, such as mice, cattle, and humans.

Researchers have determined that specific RNA-binding proteins can direct the transport of mRNAs to subcellular domains distant from the chromatoid bodies, thereby reducing the likelihood of intercellular exchange of these molecules via cytoplasmic bridges. The majority of GIMs (approximately 79%) are represented by mRNAs; however, other types of RNAs exhibiting genoinformational characteristics have also been identified in spermatid cells. The authors predicted that such genes would be more actively expressed in haploid round or elongating spermatids compared to diploid pachytene cells, which was later experimentally confirmed: GIM expression indeed peaked at post-meiotic stages [25]. Thus, genes expressed in spermatids are subject to stronger selective pressure compared to those active at earlier stages of spermatogenesis [27].

TLR7 and TLR8 belong to the class of nucleic acid (NA)-sensing receptors and possess a highly conserved structure, including a leucine-rich repeat (LRR) domain, a transmembrane (TM) domain, and a Toll/interleukin-1 receptor (TIR) homology domain. Both receptors have two ligand-binding sites: one for nucleosides and another for oligoribonucleotides [28].

Despite their overall structural similarity, TLR7 and TLR8 exhibit distinct ligand preferences: TLR7 predominantly binds guanosine, whereas TLR8 prefers uridine. Nucleoside binding occurs at the first site, forming unstable dimers that rapidly dissociate without additional stabilization. The active receptor conformation is achieved only upon simultaneous binding of nucleosides and oligoribonucleotides, leading to sustained activation of the signaling cascade [28].

It is important to note that nucleic acid degradation products, such as 2′-deoxyguanosine and 8-hydroxydeoxyguanosine, are also capable of activating TLR7 in the presence of single-stranded RNA, indicating a potential role for TLR7 in sensing endogenous cellular damage signals [29].

A critically important step in the activation of TLR7/8 is their proteolytic maturation. Specific proteases functioning within endosomal compartments cleave the structural Z-loop in the LRR domain. This cleavage removes steric hindrances to dimerization, allowing the formation of the active receptor conformation. Notably, even after cleavage, the N- and C-terminal fragments remain stably associated through extensive protein–protein interactions and, in the case of TLR7, a unique disulfide bond [30].

Activation of TLR7/8 is strictly dependent on the subcellular localization and maturation state of the receptors, which prevents their uncontrolled activation outside of endosomal compartments and minimizes autoimmune responses. Moreover, it has been shown that even double-stranded small interfering RNAs (siRNAs) can activate TLR7, underscoring the key role of nucleic acid degradation processes in their activation mechanisms.

Thus, activation of TLR7/8 represents a tightly regulated process that depends on both cooperative ligand interactions and proteolytic receptor cleavage, mechanisms that are critical for their functional activity in spermatogenic cells. It is known that signaling crosstalk with inhibitory effects can occur among different TLRs; for example, TLR8 can inhibit TLR7 and TLR9, while TLR9 can inhibit TLR7 [31]. We hypothesize that these cross-regulatory effects, along with the observed partial activity of TLR8 in spermatozoa, may be related to the peculiarities of its maturation and expression.

TLR8 with an intact Z-loop remains functionally inactive, as this structure creates a steric hindrance to the dimerization necessary for initiating the signaling cascade. Only after proteolytic cleavage of the Z-loop does dimerization become possible, relieving autoinhibition and activating the receptor [32]. In spermatozoa, where proteolytic activity may be reduced or absent, TLR8 likely fails to achieve functional maturation, which explains the lack of signaling activity.

Furthermore, TLR8 functionality may be influenced by alternative splicing and polymorphisms. In humans, the *TLR8* gene encodes at least two mRNA variants, TLR8v1 and TLR8v2, which differ in their N-terminal region: TLR8v1 contains an additional 19 amino acids. A missense polymorphism A1G (rs3764880) has also been described, resulting in a truncated isoform of TLR8v2 [33] that may alter the receptor’s ligand sensitivity or dimerization capacity. These features likely contribute to species-specific differences in TLR8 expression and activity in germ cells.

## 5. Conclusions

The study by Murat et al. [34], which provided a single-nucleus RNA-seq resource encompassing the testes of ten mammalian species and one bird, enabled comparative analysis of gene expression in spermatogenic cells across species. This research further confirmed the specific expression of TLR7/8 in murine species, particularly during the late stages of spermatogenesis. Assuming that expression in these late stages plays a key functional role helps explain why the method of sperm sorting via TLR7/8 activation may not be applicable in humans and other primates. To date, the efficacy of TLR7/8-dependent sperm sorting has been demonstrated in murine, caprine, ovine, and bovine. In contrast, this method proves ineffective in swine and canine. This discrepancy is attributed to a combination of species-specific biological factors, including the following:

- The expression patterns: in murine, TLR7 is predominantly expressed at the elongated spermatid stage, a period when functional differences between X- and Y-spermatozoa may already manifest. In swine and canine, either stage-specific expression is absent, or TLR7/8 expression levels are insufficient to initiate a signaling response.
- The proteolytic maturation of receptors: activation of TLR7/8 requires cleavage of the Z-loop within the LRR domain, enabling receptor dimerization. In the absence of proteolytic activity, as is presumed in swine and canine, the receptors remain inactive and fail to initiate the signaling cascade.
- The mRNA distribution via cytoplasmic bridges: in many species, haploid spermatids exchange molecular information through cytoplasmic bridges, which tend to minimize differences between X- and Y-cells. However, certain genes, referred to as genoinformative markers (GIMs), can partially evade this exchange. These genes are more actively expressed during the late stages of spermatogenesis and may contribute to phenotypic differences between X- and Y-spermatozoa. While this mechanism could potentially explain species-specific differences in sorting efficiency, including the limited success observed in swine and canine, further molecular studies are required to confirm such associations.
- The alternative splicing and polymorphisms. In humans, *TLR8* exists in multiple splice variants, one of which (TLR8v2) may encode a truncated protein. Such variations potentially reduce the receptor’s capacity for dimerization and activation. As a result, the application of TLR7/8-mediated sperm sorting is feasible only under strictly defined conditions, including receptor expression at post-meiotic stages, their full proteolytic activation, and the absence of intercellular equilibration compensatory mechanisms.

These findings underscore the necessity of preliminary molecular validation prior to adapting the technology to new species. Future research should focus on detailed mapping of TLR expression in germ cells of various species, including swine and canine, as well as on investigating the mechanisms of maturation and intracellular localization of these receptors. Such an approach will enable a more precise determination of the technology’s applicability and contribute to the development of novel strategies in reproductive biotechnology and sex ratio management.

Enormous economic benefits should be expected, particularly in the sheep and goat industry, where the demand for female offspring is exceptionally high in the dairy sector. The birth of male offspring brings significant economic losses to farmers. Additionally, in dairy sheep and goat breeding, the production of many male offspring, whose fattening is a significant burden for the farmer, contributes to the use of unethical practices by some farmers. Thus, optimization and implementation of the method of sexing sperm in conjunction with the use of artificial insemination of sheep and goats would solve many pressing problems, including ethical ones.

## Figures and Tables

**Table 1:** TLR7/8 localization on X-sperm cells.

Source	Specie	TLR7 Localization	TLR8 Localization	Expression Pattern
Umehara et al., 2019 [4]	Murine	Tail region of X-sperm	Midpiece of X-sperm	Both TLR7 and TLR8 expressed; X-sperm enriched for TLR7/8
Ren et al., 2021 [5]	Caprine	Entire tail	Connecting and midpiece regions	Both receptors are active and cooperatively reduce ATP levels in X-spermatozoa.
Pan et al., 2025 [6]	Canine	Tail and post-acrosomal region	Not detected	Only TLR7 detected; TLR8 absent in canine spermatozoa

**Table 2:** Successful implementation of the TLR7/8 activation method in mammals.

Source	Culture Medium	Specie	Results	Agonist Concentration	Sex Gamete Detection Method
Umehara et al., 2019 [4]	R848 + mHTF containing 2 mM glucose	Murine ^1^	Y-sperm: >90%	0.3 μM	PCR
Hou et al., 2024 [14]	dsRNA-40/cholesterol + HTF	Murine ^1^	Y-sperm: 71.59% ± 3.73% X-sperm: 79.48% ± 1.44%	0.3 μM	PCR
	dsRNA/cholesterol + HTF		Y-sperm: 68.17% ± 2.72% X-sperm: 60.43% ± 23.07%		
Umehara et al., 2020 [11]	R848 + mHTF containing 2 mM glucose + creatine	Murine ^1^	XY embryos: 92 ± 4.2%	0.3 μM	PCR
	R848 + mHTF containing 2 mM glucose + creatine	Bovine ^2^	XY embryos: 91.3 ± 2.8% XX embryos: 84.2 ± 5.3%	0.3 μM	PCR
Ren et al., 2021 [5]	R848 + goat semen extender	Caprine ^1^	Y-sperm: 90.50% ± 2.86% X-sperm: 80.30% ± 2.91%	1 μmol/L	Flow cytometry
Huang et al., 2022 [20]	R848 + semen extender at pH 7.4	Caprine ^1^	X-sperm: 85.62% ± 2.37%	0.2 μg/mL	PCR
Abadjieva et al., 2022 [22]	R848 + modified human tubal fluid medium	Ovine ^1^	Y-sperm: 74–78% X-sperm: 64–70%	0.3 μM	PCR

^1^—sperm used for each species were fresh prior to sorting; ^2^—sperm used for each species were cryopreserved prior to sorting.

**Table 3:** Unsuccessful implementation of the TLR7/8 activation method in mammals.

Source	Culture Medium	Specie	Results	Agonist Concentration	Sex Gamete Detection Method
Wu et al., 2023 [23]	R848 + semen extender	Swine ^1^	X-sperm: 46.49 ± 2.27% Y-sperm: 53.51 ± 2.27%	0.3 μM	PCR
Pan et al., 2025 [6]	R848	Canine ^1^	Y-sperm: 49.51 ± 1.12% X-sperm: 50.68 ± 0.98%	0.4 μM	PCR

^1^—sperm used for each species were fresh prior to sorting.

## Data Availability

Not applicable.
